# Genomic characterisation of an endometrial pathogenic *Escherichia coli* strain reveals the acquisition of genetic elements associated with extra-intestinal pathogenicity

**DOI:** 10.1186/1471-2164-15-1075

**Published:** 2014-12-06

**Authors:** Robert J Goldstone, Roman Popat, Hans-Joachim Schuberth, Olivier Sandra, I Martin Sheldon, David GE Smith

**Affiliations:** Institute for Infection, Immunity and Inflammation, College of Medical, Veterinary and Life Sciences, University of Glasgow, Glasgow, G12 8QQ UK; Centre for Immunity, Infection and Evolution, School of Biological Sciences, University of Edinburgh, Edinburgh, EH9 3JT UK; Immunology Unit, University of Veterinary Medicine, Bischofsholer Damm 15, 30173 Hannover, Germany; INRA, UMR1198 Biologie du Développment et Reproduction, Jouy-en-Josas, France; ENVA, Maisons Alfort, France; Institute for Life Sciences, School of Medicine, Swansea, SA2 8PP UK; Moredun Research Institute, Edinburgh, EH26 0PZ UK

**Keywords:** Comparative genomics, Evolution, Niche expansion

## Abstract

**Background:**

Strains of *Escherichia coli* cause a wide variety of intestinal and extra-intestinal diseases in both humans and animals, and are also often found in healthy individuals or the environment. Broadly, a strong phylogenetic relationship exists that distinguishes most *E. coli* causing intestinal disease from those that cause extra-intestinal disease, however, isolates within a recently described subclass of Extra-Intestinal Pathogenic *E. coli* (ExPEC), termed endometrial pathogenic *E. coli*, tend to be phylogenetically distant from the vast majority of characterised ExPECs, and more closely related to human intestinal pathogens. In this work, we investigate the genetic basis for ExPEC infection in the prototypic endometrial pathogenic *E. coli* strain MS499.

**Results:**

By investigating the genome of MS499 in comparison with a range of other *E. coli* sequences, we have discovered that this bacterium has acquired substantial lengths of DNA which encode factors more usually associated with ExPECs and less frequently found in the phylogroup relatives of MS499. Many of these acquired factors, including several iron acquisition systems and a virulence plasmid similar to that found in several ExPECs such as APEC O1 and the neonatal meningitis *E. coli* S88, play characterised roles in a variety of typical ExPEC infections and appear to have been acquired recently by the evolutionary lineage leading to MS499.

**Conclusions:**

Taking advantage of the phylogenetic relationship we describe between MS499 and several other closely related *E. coli* isolates from across the globe, we propose a step-wise evolution of a novel clade of sequence type 453 ExPECs within phylogroup B1, involving the recruitment of ExPEC virulence factors into the genome of an ancestrally non-extraintestinal *E. coli*, which has repurposed this lineage with the capacity to cause extraintestinal disease. These data reveal the genetic components which may be involved in this phenotype switching, and argue that horizontal gene exchange may be a key factor in the emergence of novel lineages of ExPECs.

**Electronic supplementary material:**

The online version of this article (doi:10.1186/1471-2164-15-1075) contains supplementary material, which is available to authorized users.

## Background

*Escherichia coli* cause a wide variety of diseases in humans and animals, carrying a significant public health and economic burden. Most *E. coli* isolates can be grouped into 7 broad phylogroups, termed A, B1, B2, C, D, E and F, based on the distribution of a number of target genes and multi-locus sequencing typing (MLST) methods [1]. Dispersed across these phylogroups are several pathotypes, including extraintestinal pathogenic *E. coli* (ExPEC) such as uropathogenic (UPEC) and neonatal meningitis-associated (NMEC), and intestinal pathogenic *E. coli* (IPEC) including enterohaemorrhagic (EHEC), enteropathogenic (EPEC), and enterotoxigenic (ETEC) strains.

Generally, a correlative relationship can be seen between pathotypes and phylogroups - *E. coli* from phylogroups B2, D and F are predominantly associated with extraintestinal diseases [2–4], and these phylogroups are enriched with genomic elements which facilitate their pathogenesis - including iron acquisition systems [5–7] and adhesins [8] - often housed on mobile genomic islands encoded in the chromosome (pathogenicity islands, PAIs), or contained within plasmids [9]. In contrast, *E. coli* from phylogroup A, B1, C and E, are less commonly associated with extraintestinal disease [2], although there may be some geographic variation to this trend [10]. These *E. coli* are more typically associated with intestinal commensalism or disease, and include the EHEC O157:H7 group (phylogroup E) infamous for several deadly outbreaks across the globe [11–14], the recent European epidemic O104:H4 group (phylogroup B1) [15], and ETEC O78:H11 str. H10407 (phylogroup A) [16]. Counter-intuitively, *E. coli* from classical ExPEC groups are also frequently isolated as human intestinal commensals, and successfully compete with resident intestinal microflora to colonise the human gut in the absence of gastro-intestinal disease [17, 18]. They may constitute the predominant faecal commensal *E. coli* type in a significant proportion of healthy humans [2, 17], and many of the PAIs which impact on ExPEC disease also function as intestinal colonisation fitness factors [19–21].

This general trend of phylogroup/pathotype association is not without exception, and isolates belonging to a particular phylogroup can exhibit the pathogenicity more typical of another. For example, one of the most closely studied intestinal pathogens, EPEC O127:H6 str. E2348/69, belongs to phylogroup B2 [22] yet houses the *lee* type 3 secretion system (T3SS) [23]; a virulence factor typically associated with EHEC from phylogroup E. Like many of the ExPEC PAIs, the T3SS is conferred on a mobile genetic element, which has facilitated the dissemination of *lee* and the attaching and effacing phenotype amongst disparate strains [24]. Similarly to the transmission of the T3SS to E2348/69, a previous investigation of closely related APEC and UPEC strains from phylogroup C identified a virulence plasmid in these strains more usually associated with typical ExPECs [25]. These isolates, which may be considered ‘pathotype switchers’, can offer an invaluable resource for investigating the genetic basis for ExPEC disease as they illuminate those components which, when acquired into an ancestrally non-ExPEC genome, may engender disease.

In this paper, we investigate the genomic evolution of extraintestinal pathogenesis in an isolate of *E. coli* termed MS499. This phylogroup B1 isolate was collected from clinical bovine metritis and can recreate extra-intestinal disease in a mouse model of infection [26]. Using a range of bioinformatic approaches, we have identified regions of the MS499 genome that are much more frequently associated with the genomes of extraintestinal pathogens from phylogroups B2, D or F, than they are with other isolates from phylogroup B1. We suggest that this ExPEC-associated DNA within the genome of MS499 has been horizontally acquired during its recent evolutionary history, and we speculate that the recruitment of this DNA into the MS499 genome has expanded the niche of this isolate, and allows it (alongside related ST453 isolates) to cause extraintestinal disease. The acquired DNA disproportionately encodes several functional classes of genes, including those involved in adhesion and those directed to the outer membrane or extracellular space. These data help illuminate key components in the genetic basis for extraintestinal pathogenesis in *E. coli*, and demonstrate the adaptability of this species in its evolution to exploit multiple environments.

## Results and discussion

### MS499 is a member of an emerging ExPEC lineage

*E. coli* strain MS499 (ST453) was isolated from a case of bovine metritis, and previously shown to reproduce extra-intestinal disease in a mouse model of intrauterine infection [26]. In light of the ability for MS499 to cause extraintestinal disease in animals, we investigated whether *E. coli* similar to MS499 had been isolated from other extraintestinal infections. We searched the literature for reference to this sequence type (ST453) and found seven recent publications describing eight ST453 isolates, five of which are isolates from human urinary tract infections across the globe (Table 1). The remaining three isolates, alongside two further unpublished isolates found in the *E. coli* MLST database (Ecoli-CHU-52 and Ecoli-CHU-24), are isolated from the faeces of humans or animals in the absence of disease. These data revealed a striking mirror of the epidemiological profile of traditional ExPECs (a combination of extraintestinal isolates and human intestinal commensals) in this phylogroup B1 lineage, and demonstrate increasing evidence for the ability of ST453 *E. coli* to cause extra-intestinal disease. Since this group is outwith the previously dominantly represented, and well characterised, ExPEC lineages from phylogroups B2, D and F, we speculate that ST453 may represent a distinct and potentially emerging ExPEC lineage.

**Table 1:** **Isolated ST453 strains and their source of isolation**

Strain	Country	Source	Pathotype	Reference
*E. coli* 28	Brazil	Human	UPEC	[27]
ESBL116	Netherlands	Human	UPEC	[28]
HMMC097	Brazil	Human	UPEC	[10]
IMT19121	Germany	Rat	Faecal	[29]
U2183	Germany	Human	UPEC	[30]
Not given	Portugal	Seagull	Faecal	[31]
Not given	China	Human	UPEC	[32]
Not given	China	Human	Faecal	[32]
*Ecoli-CHU-52	France	Human	Faecal	CHU J. Minjoz Besançon
*Ecoli-CHU-24	France	Human	Faecal	CHU J. Minjoz Besançon

*Unpublished isolate present in the University College Cork MLST database.

ST453 *E. coli* previously isolated from humans and animals. Five of the eight isolates are from urinary tract infection of humans (UPEC), while the remaining three are faecal isolates from humans and animals. Two further unpublished isolates present in the MLST database (University College Cork, marked with an asterisk) have been isolated from humans in the absence of disease.

### Overview of the MS499 genome

Our investigation of the MS499 genome sequence [33] (accession number JDRV01) revealed that, in addition to the chromosome, this sequence contained several contigs which exhibited marked homology to *colV*-type plasmids found in a number of ExPECs including APEC O1, S88 and several plasmids isolated from extra-intestinal pathogenic *Salmonella enterica* serovar Kentucky isolates (Additional file 1), which have been proposed to underpin their virulence [34, 35]. A similar plasmid was also recently described in an ExPEC isolate from phylogroup C [25], indicating that the putative MS499 plasmid could also play a role in virulence. Indeed, many of the traits associated with ExPECs can be conferred when similar plasmids have been experimentally introduced into non-pathogenic *E. coli* [36]. Among the fitness, colonisation and virulence factors encoded on the putative MS499 plasmid are those which may provide resistance against several antibiotics, including beta-lactams, tetracyclines and macrolides. The presence of these plasmid sequences in MS499, and similar plasmids in the genomes of other ST453, isolates may explain the some of the antibiotic resistance previously observed in these strains [26–29, 31, 32].

Our preliminary investigation of the MS499 chromosome revealed several features in the genome of this isolate which we did not anticipate, and could be related to the ability for MS499 to cause ExPEC infection. These included the Yersiniabactin iron uptake system, implicated in the virulence of a number of ExPECs [37], the Pix fimbrial locus, previously identified in the UPEC strains X2194 and 536 [38, 39], and a group 3 extracellular polysaccharide capsule locus with similarity to that described in the sepsis-associated isolate CP9 [40, 41].

### Regions of the MS499 genome show association with ExPECs

**Figure 1:**
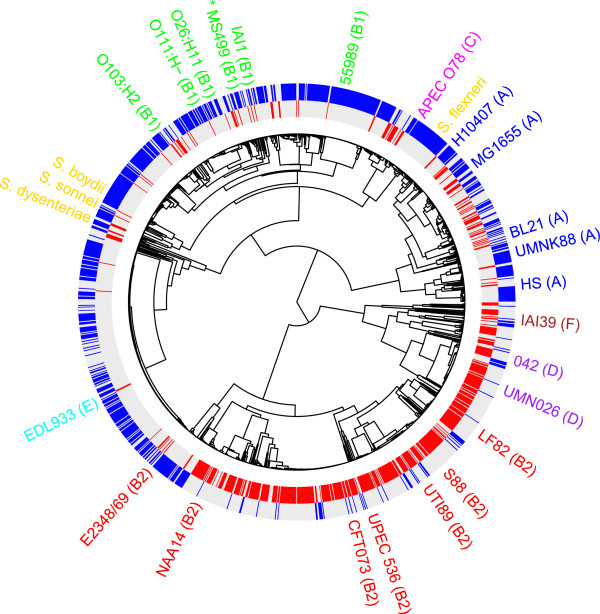
**The phylogenetic structure of** ***Escherichia coli.*** The phylogenetic relationship between 1581 sequenced *E. coli* genomes based on the concatenated sequence of 159 core genes as estimated by maximum likelihood, and the distribution of an intestinal (blue) versus extra-intestinal (red) source of isolation. Isolates coloured grey in both channels have either been isolated from a non-intestinal or extra-intestinal source, or no data is available for their source of isolation. The position of MS499 in the dendrogram is marked with an asterisk. A bootstrapped tree using a subset of these isolates is included in Additional file 3.

In light of the fact that ST453 contained several examples of isolates from extra-intestinal infection, we speculated that, as a representative of ST453, MS499 may possess genes which enable it to behave more similarly to typical ExPECs within phylogroup B2, D or F, than to members of its phylogenetic family (phylogroup B1). We reasoned that regions of the MS499 genome that are more frequently found in ExPECs than within phylogroup B1 organisms would be likely to contain candidate genes to be involved in the MS499 ExPEC phenotype. To investigate this, we sought to compare the MS499 genome to two groups of sequences; one of typical ExPEC genomes, and the second of genomes from the phylogroup B1 relatives of MS499. To populate these groups with a sufficient number of sequences for robust comparative analysis, we elaborated the phylogenetic relationship between 1581 *E. coli* genome sequences available in the NCBI sequence database at the outset of the study, and approximated their isolation source using the data available at NCBI (Figure 1). We partitioned 414 genomes into the ExPEC group, and 365 genomes into the B1 group - further details of the genomes assigned to each group are shown in the table in Additional file 2. Due to the large number of genomes in this analysis bootstrapping of this tree was not possible, so a subset of these sequences were taken for phylogenetic analysis with bootstrapping in order to support the branching pattern, which showed strong support for our tree (Additional file 3).

**Figure 2:**
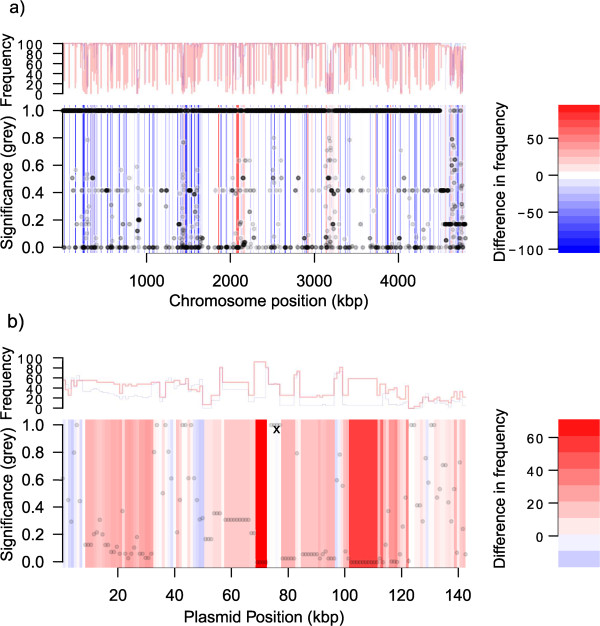
**The association between the MS499 genome and the phylogroup B1 or ExPECs groups.** The scale to the left of the image describes the colours which indicate a difference in frequency between the phylogroup B1 group (−100 to < 0, blue) and ExPEC group ( > 0 to +100, red). These colours are used to represent query windows across the length of the MS499 chromosome **(a)** and plasmid **(b)**, where regions more frequently found in ExPECs than B1s are red, regions of no difference between the two groups are white, while regions more frequently found in B1s than ExPECs are blue. Significance values on the y-axis are the results of two-tailed Fisher’s exact test to determine whether a query window is significantly associated with either group, and the results for each query window are represented on the graph by semi-transparent grey circles (where circles overlap, these appear darker). Regions of DNA which appear white in the main panel do not differentiate between query windows which are uniformly common or uniformly rare, therefore the line graphs presented above the main panels track the frequency of each window in the B1 group (blue line) and ExPEC group (red line) independently. These data indicate that approximately 4% of the MS499 chromosome (within 52 contiguous regions) and 52% of the putative MS499 plasmid (within 10 contiguous regions) are significantly associated with the ExPEC group (p = < 0.0000021 for the chromosome, < 0.00007 for the plasmid).

Rather than focusing entirely on previously-defined genes for our comparative analysis, we performed an unbiased analysis in order to identify any region of the MS499 genome which displayed a significantly greater association with sequences in the ExPEC group than those within the phylogroup B1 group. To do this we investigated the frequency of homologous sequences to 1 kb ‘windows’ of the MS499 genome in the B1 or ExPEC sequence groups. The resulting data allowed us to calculate the difference in frequency between the two groups, which is expressed on a scale from −100 to +100, representing windows more frequent in B1 than ExPECs (− 100 to < 0), regions of no difference between the groups (0) and windows more frequent in ExPECs than B1 ( > 0 to +100). The main panels in Figure 2 represent this frequency difference along the length of the MS499 chromosome (a) and plasmid (b), according to the scale presented to the right of the image. The grey circles on the main panels represent results of a statistical analysis (Fisher’s exact test, scale shown on the y-axis) to identify windows significantly associated with either group. Since regions of no difference (white) do not discriminate between regions of shared commonality (i.e. both groups contain the query window at high frequency) or shared rarity (i.e. both groups contain the query window at low frequency), the top panel for the chromosome and plasmid tracks the total frequency in the B1 group (blue line) and ExPEC group (red line) independently.

The data in Figure 2 revealed some striking features of the MS499 genome, with numerous regions clearly associated with either the B1 or the ExPEC group. Our analysis of these data showed that approximately 12.5% of the MS499 chromosome is significantly enriched in the B1 group - this result was anticipated, since MS499 is a phylogroup B1 strain. These regions encode several metabolic loci (including the *hpa* and *paa* aromatic hydrocarbon catabolism loci and several other putative carbon utilisation pathways) and a number of genes involved in the production of extracellular structures including fimbrial and autotransporter genes. Conversely, we found a surprisingly large fraction (approximately 4%) of the chromosomal windows to be significantly enriched in the ExPEC group compared to the B1 group. Several prominent ExPEC associated regions are visible in the chromosome of MS499, particularly a block at approximately 2100 kbp, which is the product of 32 consecutive windows which are strongly associated with the ExPEC-group in contrast to the B1 group, and coincides with the Yersiniabactin siderophore system locus. In addition, the MS499 plasmid appears predominantly ExPEC-associated, with approximately 54% showing a strong, significant association with ExPECs. A full list of B1-enriched and ExPEC-enriched genes is included in Additional file 4).

### MS499 is more ExPEC-like than is typical for other B1 isolates

**Figure 3:**
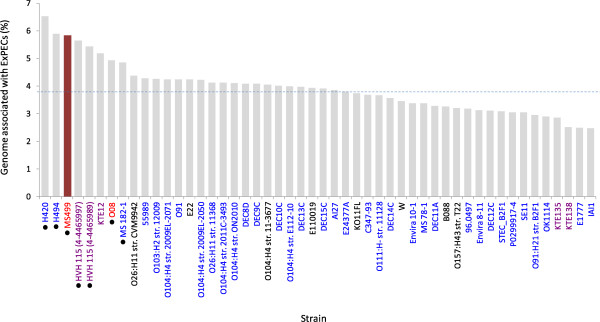
**The percentage genomic association with the ExPEC group compared with the phylogroup B1 group in a range of phylogroup B1 genomes.** On average, 3.9% of a phylogroup B1 genome contains DNA which is more frequently found in ExPECs (dotted line). By contrast, 5.8% of the MS499 genome (highlighted) is more frequently found in ExPECs - a 33% increase on the average. Isolates which have been recovered from intestinal sites are coloured blue, while those which have been recovered from extra-intestinal sites are coloured red (isolates where the source is unknown are black). Isolates belonging to ST453 are annotated with a black circle next to the name. This detail shows that all the identified ST453 strains contain more than the average quantity of ExPEC-associated DNA that is typical for phylogroup B1 genomes.

In total, we calculated that 5.8% of the MS499 genome (chromosome + plasmid) is significantly more prevalent in the genomes of ExPECs compared to phylogroup B1, which raised the question as to how typical this was for phylogroup B1 genomes in general. To investigate this, we investigated the amount of ExPEC-associated DNA found within 50 other genomes across the spectrum of phylogroup B1*,* and calculated this as a percentage of their genome length (Figure 3).

Figure 3 reveals that MS499 encodes an atypically high percentage of ExPEC-associated DNA, in comparison to the average of what may be expected for a phylogroup B1 genome. From the 50 phylogroup B1 genomes investigated, MS499 ranks third in the greatest amount of ExPEC-associated DNA contained within a genome. Furthermore, the two higher ranking isolates (H420 and H494), along with other top ranking isolates (HVH_997, HVH_989, H494, MS182-1 and O08), are all ST453 strains very closely related to MS499 (Additional file 5), whilst O08 was also isolated from extra-intestinal infection [42]. The increased amount of ExPEC-associated DNA in the MS499 genome relative to the average for phylogroup B1 equates to approximately 100 kbp.

### Gene families over-represented in the MS499 ExPEC-associated DNA

The surprising association of approximately 5.8% of the MS499 genome with ExPECs led us to investigate the function of the genes within these regions, as we presume that these directly influence the ability for MS499 to cause extraintestinal disease. We identified 170 chromosomal and 67 plasmid-borne genes which localised to the ExPEC-associated windows (Additional file 4). Key genes which have previously been implicated as important to ExPEC virulence and, we speculate, are crucial to the MS499 ExPEC phenotype – are listed in Table 2. Unexpected among these was type I fimbriae which, although found widely in *E. coli*, have been linked with ExPEC pathogenicity [43, 44]. The *fim* genes were, surprisingly, underrepresented in phylogroup B1 genomes compared with ExPEC genomes, hence, they emerged as a significant correlate.

**Table 2:** **Key ExPEC-like genes in the genome of MS499**

Gene/group	Function	Reference
Yersiniabactin	Siderophore iron acquisition system	[37]
*kpsS*	Capsular polysaccharide biosynthesis	[40]
Fim	Type I fimbrae for adhesion to surfaces	[43]
Sit (p)	Iron / Manganese transport	[45]
Salmochelin (p)	Siderophore iron acquisition system	[6]
Aerobactin (p)	Siderophore iron acquisition system	[7]

Key genes present in the genome of MS499 which have previously been implicated in extra-intestinal pathogenesis including iron acquisition systems, adhesive factors and capsule. Factors encoded on the MS499 plasmid are labelled (p).

**Figure 4:**
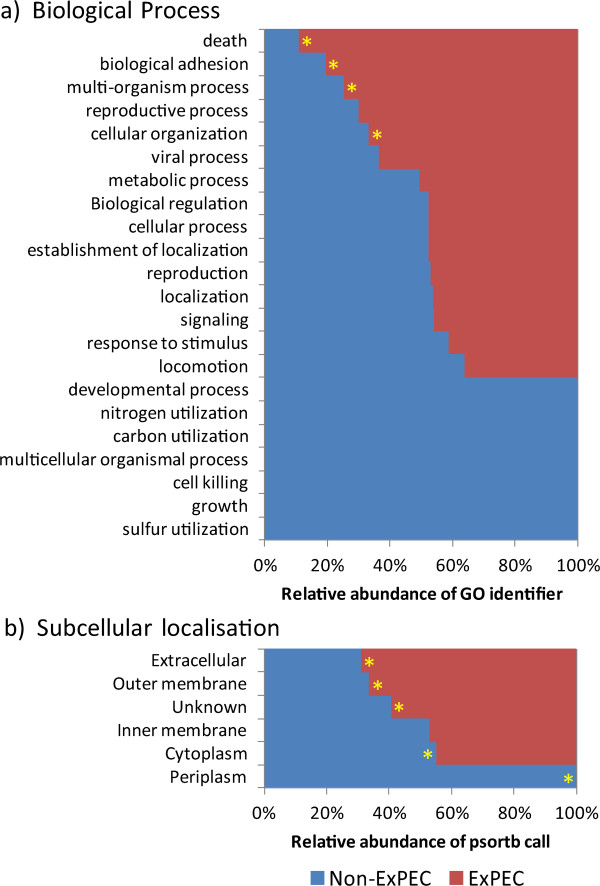
**A comparison of the relative abundance of biological process Gene Ontology classifications (a) and subcellular localisation predictions (b) for the MS499 non-ExPEC genome (blue) versus the MS499 ExPEC-associated genome (red).** These data show that the ExPEC-associated DNA in MS499 disproportionately carries genes related to several processes which may be important for its ExPEC lifestyle, including **(a)** genes related to biological adhesion and the coordination of populations (multi-organism process) as well as **(b)** genes directed to the outer membrane of extracellular space. In total, GO annotations were recoverable for 3663 genes. Statistical significance, annotated by an asterisk on the over-represented side, was determined by Fisher’s exact test (p = < 0.05).

In addition to the role for the characterised ExPEC determinants, we speculated that the ExPEC-associated genes may enrich MS499 for certain functional classes of protein and facilitate extraintestinal pathogenesis. To investigate this, we examined the ‘biological process’ Gene Ontology (GO) annotations [46] (Figure 4a), and pSORTb sub-cellular predictions [47] (Figure 4b), for the ExPEC-associated genes compared with other genes in the MS499 genome (Figure 4a).

This analysis revealed several functional classes and localisations of genes to be over-represented in the ExPEC-associated DNA of MS499 (Figure 4, red; significant differences marked with an asterisk) compared to that which constitutes the non-ExPEC DNA (Figure 4, blue). Statistical investigation (Fisher’s exact test, p < 0.05) indicate that genes involved in biological processes including “death” (GO:0016265) (typically phage-related genes), “biological adhesion” (GO:0022610), “multi-organism processes” (GO:0051704), and “cellular organisation” (GO:0071840), are all significantly over-represented in the MS499 ExPEC-associated DNA. For the predicted subcellular localisation, these data indicate that genes whose products are directed to the outer membrane or extracellular space are also over-represented in the ExPEC-associated genes, genes localised to the cytoplasmic membrane show no difference, while genes localised to the cytoplasm or periplasm are significantly under-represented.

The gene classes contained disproportionately in the MS499 ExPEC-associated DNA are reflective of many of traits associated with ExPEC disease, including adhesins and secreted factors [17], while the over-representation of genes related to death is indicative of the enrichment of phage/prophage within the ExPEC-associated DNA, which may have carried ExPEC-associated traits into the genome of MS499. Both the number of ExPEC-associated genes in the genome of MS499 and the range of functional classes that these genes fall into suggests ExPEC pathogenesis in MS499 is unlikely to be conferred by a narrow subset of genes, but is more likely to be a complex trait resulting from the incremental contribution of a number of interlinked processes.

### MS499 acquired much of the ExPEC-associated DNA via horizontal transfer

Several of the ExPEC-associated features in the genome of MS499, including the plasmid and the Yersiniabactin iron uptake system are well characterised mobile genetic elements [48, 49], suggesting that some of the ExPEC-associated DNA present in the MS499 genome may be a result of recent horizontal transfer. To investigate this, we reasoned that by profiling the ExPEC-associated regions in the MS499 genome for their presence or absence in the sequences of its closest relatives (for which genomes were available), we could identify the regions which have been acquired over the evolution of this strain. Our broad phylogenetic estimate (Figure 1) suggested that 14 genome sequences present in NCBI were highly similar to MS499. These relationships were confirmed by examining SNPs in the core genome of these sequences using PanSeq [50] (Additional file 5).

**Figure 5:**
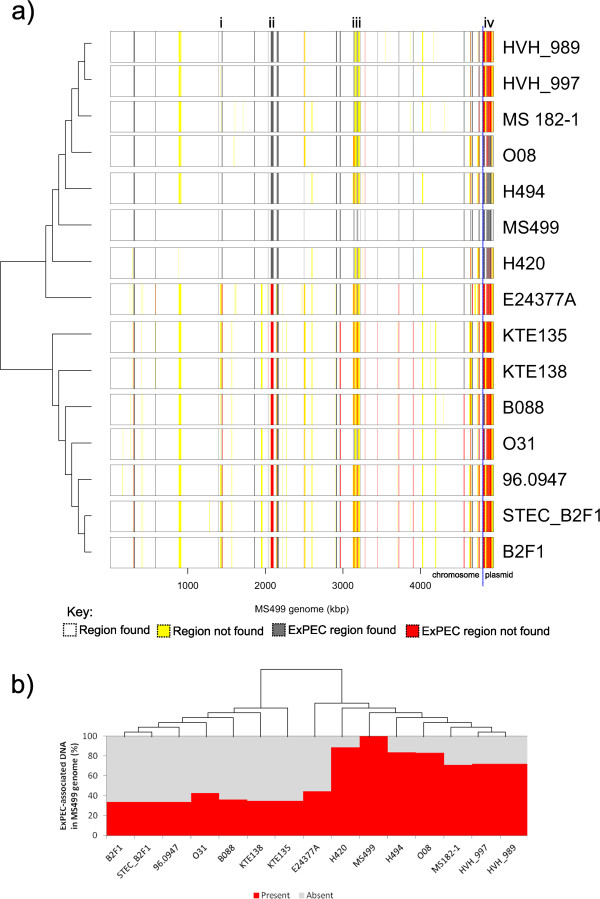
**Diagrammatic representation of the similarity between the MS499 genome and its close relatives in reference to the ExPEC-associated DNA found within its genome.** We compared the MS499 genome sequence to its close relatives to investigate the structure and variation in the ExPEC-associated regions in the MS499 genome **(a)**. Regions of the non-ExPEC associated DNA in the MS499 genome which are also found in the isolate are coloured white, regions of this DNA which are not found in the isolate are coloured yellow. ExPEC-associated DNA which is found in the isolate are coloured grey, and ExPEC-associated DNA which is not found in the isolate are red. Several regions of clear variation between the MS499 genome and the related isolates are evident (listed i – iv), within which the ExPEC-associated DNA appears to be concentrated. We then investigated the proportion of the MS499 ExPEC-associated DNA which could be found in the genomes of these relatives **(b)**, which showed that closer phylogenetic relatives of MS499 tend to encode more of the ExPEC-associated DNA in common with MS499 than do other, more distantly related isolates. The dendrograms presented alongside each panel are linear representations of the phylogeny in Additional file 7: Figure S4 with bootstrap values removed.

These data supported the validity of our phylogenetic estimate, as the topology of the tree shown in Additional file 5 was very similar to the local topology around MS499 in the tree within Figure 1. Using these genome sequences as close relatives of MS499 we identified regions of the MS499 chromosome or plasmid which were absent in each of these close relatives. We then combined this data with the information on the ExPEC-associated DNA in MS499 to visualise four categories, as shown in Figure 5 (a): (1) regions of the backbone MS499 genome which are found in a relative (white), (2) regions of the backbone genome which are not found in a relative (yellow), (3) ExPEC-associated regions which are found in a relative (grey), and (4) ExPEC-associated regions which are not found in a relative (red). This analysis revealed several hotspots in the MS499 genome which varied in the other genomes, within which the ExPEC-associated DNA appears concentrated. For example, MS499 shares approximately 91% of its total genome with the ETEC strain E24377A, however the 9% that is not found in the E24377A genome accounts for over 55% of the ExPEC-associated DNA present in the MS499 genome. It also appears that more immediate relatives of MS499 (other ST453 strains such as H420, H494 and O08) tend to encode more of the ExPEC-associated DNA in common with MS499 than more distant relatives (such as B2F1 and B088). To investigate this further, we calculated the proportion of ExPEC-associated DNA in the MS499 genome which is present in the genomes of these relatives (Figure 5b). This analysis revealed a clear trend for more closely related isolates to MS499 to share more of the ExPEC-associated DNA with this isolate than less closely related ones, consistent with the hypothesis that MS499 has acquired the ExPEC-associated DNA over the course of its recent evolution and since divergence from E24377A.

**Figure 6:**
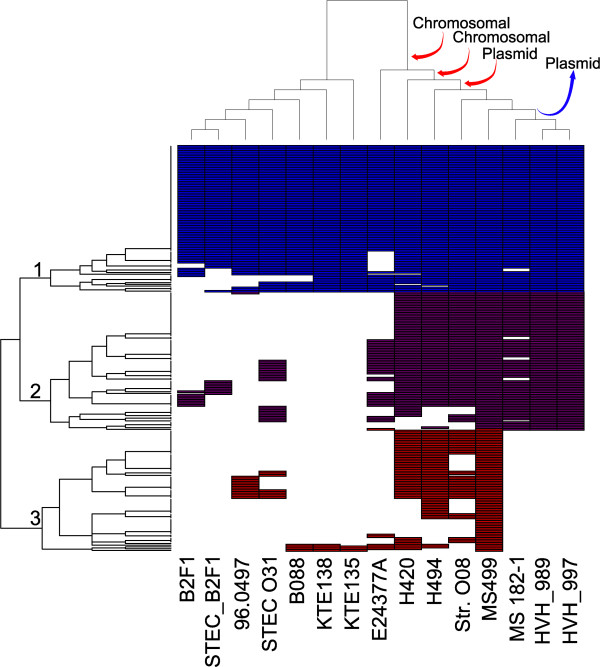
**Clustering of the distribution of ExPEC-associated genes in close relatives of MS499.** The distribution of ExPEC-associated genes from MS499 are clustered according to the row dendrogram. Three broad groups of genes can be resolved from this analysis: (1) chromosomal genes which appear to be ancestral to the MS499 lineage, (2) chromosomal genes which are present in a subset of 7 genomes in the group, including MS499 and its six closest relatives, and, (3) plasmid genes present in MS499 and its three most proximal phylogenetic relatives. The column dendrogram has been ordered according to the phylogenetic relatedness between the strains as show in Additional file 5. From these relationships, a model can be proposed where the MS499 acquired some chromosomally encoded ExPEC genes in two stages, with the acquisition of the plasmid occurring subsequently (red arrows). This model suggests that the plasmid has been lost in the lineage leading to MS 182–1 and the two HVH strains (blue arrow).

To build a model of the stages in the acquisition of the ExPEC-associated DNA by MS499, we investigated the distribution of the ExPEC-associated genes in the genomes of the close relatives, and performed clustering based on the distribution of genes between the different genomes. This clustering, shown on the row dendrogram in Figure 6, is compared against the phylogenetic relatedness between the isolates, shown on the column dendrogram, which is taken from the phylogenetic analysis shown in Additional file 5.

This analysis supports the hypothesis that a large proportion of the ExPEC-associated genes within the MS499 genome have been acquired by horizontal gene transfer during its recent evolutionary history. The horizontal clustering in Figure 6 suggests three distinct groups: (1) genes which appear ancestral to the linage leading to MS499, as they are found in the majority of this set of genomes (with putative loss in some genomes), (2) chromosomally encoded ExPEC genes, some of which are found in E24377A, but most are found only in 6 of the closest relatives of MS499 and (3) a group of mainly plasmid encoded genes which are found only in three of the most phylogenetically proximal isolates to MS499. Comparison with the phylogenetic relatedness (column dendrogram) suggests that these ExPEC genes have been acquired in successive stages, with two transference events leading to the combination of chromosomal genes, and a subsequent acquisition of the plasmid. Interestingly, this analysis also suggests this plasmid to be instable, with subsets of the plasmid encoded genes missing in strains H420, H494 and Str. O08 (which all encode a similar plasmid to MS499). Furthermore, this model suggests the plasmid may have been subsequently lost by the lineage leading to MS 182–1, HVH_989, HVH_997.

Given that our data indicate that many of the ExPEC-associated genes were acquired by MS499 via horizontal gene transfer, we performed a more detailed comparison of the relationships between the sequences of these genes in MS499, and those present in other ExPECs, in order to trace the provenance of these genes. To do this we constructed phylogenetic trees for each of the genes within clusters 2 and 3 from Figure 6, and calculated the distances between the sequences found in MS499, and the cognate sequences from other ExPECs (Additional file 6).

As we expected, no single ExPEC genome contained homologues to all of the ExPEC-associated genes present in the MS499 genome, which is consistent with the hypothesis that MS499 acquired these genes in stages rather than *en bloc* in a single interaction with a donor source. Conversely, 33 of the 34 ExPEC-associated genes we postulate were acquired by the last common ancestor of MS499 and E24377A have very similar homologues in 15 closely related phylogroup B2 strains- of which the most similar is the isolate HVH 212(3–9305343). Further investigation of the HVH 212(3–9305343) genome revealed a 31 bp remnant of the missing gene (corresponding to gene 90 in Additional file 4), suggesting that this gene may have been previously present in this lineage, but has since become substantially truncated. Therefore, it is probable that a HVH 212(3–9305343)-like *E. coli* was the donor source for the complement of ExPEC-associated genes acquired by the last common ancestor of MS499 and E24377A.

Similarly, the ExPEC-associated genes putatively encoded on the MS499 plasmid can also all be found in 14 other isolates - in this case 13 originate from phylogroup B2 and one from phylogroup D, whilst the most similar genes are found in the phylogroup B2 isolate HVH 146(4–3189767). Indeed, further analysis of these additional genomes revealed several, and in particular HVH 146(4–3189767), to contain highly homologous sequences to the entirety of the putative MS499 plasmid (Additional file 7). As expected, the average distance between the MS499 plasmid gene sequences and those in HVH-146(4–3189767) is much smaller (0.01) than between the chromosomal genes shared between MS499 and HVH 212(3–9305343) (0.29), consistent with the plasmid having been acquired much more recently by the MS499 lineage. Although it is possible that, rather than the MS499 lineage acting as a recipient for the plasmid from these strains, instead MS499 served as the donor of this plasmid, we consider this scenario unlikely due to evidence for similar plasmids in a high number of isolates closely related to HVH 146(4–3189767) within phylogroup B2.

However, the provenance of the chromosomal genes acquired by the MS499 lineage subsequent to its divergence from E24377A appears more nuanced, and we could not clearly demarcate putative donor strains which could have contributed all of these genes to the MS499 genome. Some genes, such as the Yersiniabactin iron uptake locus, are highly similar to those found in phylogroup D strains KTE80, HVH 113(4–7535473), HVH 140(4–7535473) and HM69, whilst others, for example the phosphoglyerate transport (*pgt*) locus, are much more similar to cognate genes found in phylogroup B2 strains KTE180, HVH 42(4–21000061) and HVH 126(4–6034225). This suggests that at least 2 separate interactions have occurred which have resulted in the specific arrangement of these ExPEC-associated genes in the MS499 genome, however we cannot rule out the possibility that a single donor strain existed which is not represented in the panel of sequences we used.

## Conclusions

These results suggest that MS499 and ST453 *E. coli* represent an emerging extraintestinal pathogen with world-wide presence. Strikingly, the isolates from this sequence type share an epidemiological profile very similar to ExPECs, with a low incidence of intestinal pathogenic isolates combined with a high incidence of both intestinal commensal and extra-intestinal pathogenic variants. Our present work suggests this is likely to be underpinned by regions of the genome which are reminiscent of extraintestinal pathogenic *E. coli* in phylogroup B2, D and F, which are much less likely to be found amongst other phylogroup B1 *E. coli*. These regions disproportionately encode traits, including iron acquisition systems and adhesins, which are crucial for the diseases caused by classic ExPECs. Furthermore, our analyses indicate that over half of the total ExPEC-associated DNA within the MS499 lineage has been acquired since its divergence from E24377A, which our data indicate to be the closest diarrheagenic relative to the ST453 group. This DNA, including the chromosomally encoded yersiniabactin locus, *kpsS* and the MS499 plasmid, appear to have been acquired in stages, suggestive of an iterative evolutionary process which has gradually recapitulated the ExPEC phenotype in a typically non-ExPEC background, thus allowing MS499, and potentially other ST453 isolates which share this genetic information, to colonise niches broadly unavailable to their ancestral group. These analyses help illuminate the genetic basis for extraintestinal disease, since the data reveal those factors which, when shuffled together, are sufficient to permit the emergence of a novel ExPEC lineage.

For the farm from which MS499 was isolated, ST453 was the dominant disease-associated clone present at the time of sampling [26]. However, this and another study have shown that metritis-associated *E. coli* originate from a diverse range of phylogenetic lineages, albeit with a preponderance for isolates from phylogroup B1 [51]. Although these studies found few genes to be associated with metritis *E. coli*, Bicalho and colleagues saw a modest enrichment of *fimH* carrying isolates in metritis [51], which is concordant with the differential carriage of type I fimbriae we found between ExPEC genomes and those from phylogroup B1. However, the general failure of previous studies to identify genes associated with metritis may indicate that the causative *E. coli* utilise disparate virulence strategies to colonise and cause disease in the bovine uterus, yet it also may reflect the fact that the ExPEC-associated genes we have identified in MS499 are often unrepresented in PCR based analyses of virulence gene carriage. We anticipate the results presented here will inform future population level analyses of *E. coli* associated with metritis and other atypical ExPECs, to investigate if these, or a subset of these genes, can define this economically important pathotype and be used as diagnostic or therapeutic targets.

## Methods

### Sequences used in this study

A list of the 1581 sequences used in this study, their source of isolation and phylogroup are listed in Additional file 2. All sequences were available in the public domain at the initiation of the work. All gene prediction was carried out using Prodigal [52].

Genome source information was collected from the data for each genome housed at NCBI. Unfortunately, this metadata was incomplete for the majority of the sequences (~60%), and so to supplement the available data we used the focus of the study for which the isolates were collected as a proxy for their source of isolation (for example *E. coli* bacteraemia, UTI and defensins).

### Phylogenetic estimation of *E. coli*

The nucleotide sequences for core-genes in *E. coli* were elaborated by initialising the the core gene set (CGS) as the nucleotide sequences for genes present in MG1655. At each iteration, the CGS was aligned to the next *E. coli* genome sequence using blastn [53]. Genes aligning at > 70% identity and > 80% of the length of the coding sequence were retained in the CGS for use in the next iteration. The resulting nucleotide sequences of 159 genes were extracted from the *E. coli* genomes using blastn [53], aligned by Muscle [54] and concatenated. A maximum likelihood tree was constructed under the GTR + g model via PhyML [55]. Bootstrapping proved challenging for this large tree, and so a subset of these sequences were selected from the alignment, and a tree built under the same parameters with 100 bootstrap replicates (Additional file 3). The circular phylogenetic tree and trait plot was visualised using the diversitree [56] and ape [57] packages in R. Further investigation of the paths in the tree topology to define phylogroup B1, and ExPECs within phylogroup B2 and phylogroup D isolates was were achieved using ade4 package within R [58].

### *In silico*assembly of the MS499 chromosome and plasmid

To assemble the MS499 chromosome and plasmid *in silico,* we re-ordered the recently released MS499 contigs [33] against the E24377A chromosome sequence, since our phylogenetic analysis indicated this to be the closest completely assembled relative of MS499. Overlapping, aligned contigs for MS499 were joined into supercontigs. When no overlapping sequences could be identified, the termini were compared to the E24377A sequence. When this showed the contig termini to abut each other, contigs were joined or missing bases (up to 5) were donated from the E24377A sequence. Larger sequences of missing bases were searched for in the non-aligned contigs, and when identified used to join the contigs together. Occasionally, inspection showed the contig termini clearly deviated from the E24377A reference sequence, and in these cases they were investigated by BLAST analysis [53] against all *E. coli* sequences. Contig termini with a high level of homology to insertion sequence (IS) elements were joined by the internal sequence of the IS element identified in small, unaligned contigs. Contig termini showing evidence of sequences from phage or other mobile genetic elements present in other *E. coli* were investigated for homology in unaligned contigs, and these regions joined. Through this iterative approach, we resolved a single supercontig for the chromosome of MS499. We used a similar approach to assemble contigs for the MS499 plasmid, where contigs were initially re-ordered against the sequence for pECOS88. Following this, we checked our assembly by re-mapping the Illumina reads to our supercontig using SAMtools [59] and visualised this map using Artemis, where we manually confirmed that reads overlapped continuously over all regions of the chromosome and plasmid.

### Investigating the frequency of the MS499 genome within the B1 and ExPEC groups

To investigate the association between the MS499 genome and ExPEC or phylogroup B1 groups, we used our phylogenetic analysis to identify 365 genomes in phylogroup B1 and 414 extra-intestinal pathogenic *E. coli* within phylogroups B2, D and F (Additional file 2), and each set of genomes were formatted into BLAST databases. The MS499 chromosome and plasmid were split into 1000 bp sequential windows, and each window used as the query sequence against both BLAST databases so that the number of genomes in each group which contained a subject sequence which matched the query sequence at a BLAST e-value of zero was determined. The percentage frequency of the window sequences in the groups was calculated, and the difference in frequency determined as the percentage frequency in the ExPEC group minus the percentage frequency in the B1 group. For statistical analysis, Fisher’s exact test was applied to each window, and the p value requirement for statistical significance (1% chance) was adjusted for multiple tests (p = < 0.0000021, for the chromosome- 4798 tests; p = < 0.00007 for the plasmid- 142 tests). The difference in frequency was plotted along the length of the MS499 chromosome and plasmid using the image function within R [60]. Subsequent to this, genes which overlapped with the identified ExPEC-associated regions in the MS499 genome by more than 60% of the coding sequence were identified for further analysis.

### Gene ontology annotation

To extract the Gene Ontology (GO) annotations for the MS499 genes, we downloaded the GO annotations for MG1655, E24377A and APEC O1 from UniProt-GOA [61]. We then compared the MS499 genes to each of the genes for MG1655, E24377A and APEC O1 and transferred the GO annotations from genes which shared greater than 80% identity with genes in MS499. Genes lacking annotation following this were imported into Blast2GO [62] and annotated using this program. GO annotations from both methods were then combined for subsequent analysis.

### GO and psortb annotation comparison

To compare the relative abundance of biological process (BP) GO annotations for the ExPEC genes versus the non-ExPEC genes in the MS499 genome, the BP GO annotations for each gene were traced back to their respective level 2 classification using the GOstats package within Bioconductor in R [63] . Since any given gene may have more than one GO annotation, and the respective level 2 classification of each of the annotations may be the same or different, we ensured that no level 2 classification was present in duplicate for any gene. To calculate the relative abundance of each GO category, we determined the abundance of each GO category in each group (ExPEC genes or non-ExPEC genes) and compared the abundance of each category between the two groups. The same method was used for comparing the prediction for subcellular localisation using pSORTb [47].

### Detailed sequence comparison of ExPEC genes

To perform a detailed analysis of the relationships between ExPEC-associated genes found in the MS499 genome, we probed for, and extracted, the sequence of these genes from 414 ExPEC genomes via blastn [53], using the nucleotide sequence for the gene present in MS499 as the query. The sequences were aligned by Muscle [54], and maximum likelihood trees for each gene constructed under the GTR + g model via PhyML [55]. Following this, matrices for the phylogenetic distances between the MS499 gene sequences and those found in other ExPECs were calculated via the ‘cophenetic’ function from the APE package [57] launched within R [60]. Missing genes was given a penalty distance of 100, and these distances were used to calculate which ExPEC genome encoded the most similar genes to those found in MS499.

## Availability of supporting data

This research utilised publicly available bacterial genome sequence data for which there are no ethical issues. The data sets supporting the results of this article are included within the article and its additional files. The MS499 genome is available in the DDBJ/EMBL/GenBank under the accession number JDRV00000000.

## Electronic supplementary material

Additional file 1: **Comparison of the putative MS499 with similar plasmids.** Comparison of the MS499 plasmid with similar plasmids found in other extra-intestinal pathogenic *E. coli* and *Salmonella enterica* isolates. (PDF 446 KB)

Additional file 2: **Additional information for genomes collected in this study.** Accession numbers, source of isolation and phylogroup of genomes used in the work. (XLSX 118 KB)

Additional file 3: **Phylogenetic analysis with bootstrap support.** A subset of sequences used in our phylogenetic analysis with bootstrapping to support the branching pattern. (PDF 170 KB)

Additional file 4: **Genes identified as associated with the B1 phylogroup or ExPECs.** A list of genes which were identified in the B1-associated (tab 1) or ExPEC-associated (tab 2) genome of MS499. (XLSX 147 KB)

Additional file 5: **The local phylogeny of MS499.** A phylogenetic analysis of genomes closely related to MS499. (PDF 272 KB)

Additional file 6: **Phylogenetic distances between ExPEC-associated genes.** This file contains the distances calculated between the sequences for the ExPEC-associated genes present in MS499 compared with other ExPEC genomes in which they are found. This file also includes the data which underlies the heatmap in Figure 5, which is important for understanding how the genes were subdivided into groups. The first tab in the file explains how the data is represented, the second tab contains the chromosomal genes and the third tab contains the putative plasmid genes. (XLSX 547 KB)

Additional file 7: **Homologous sequences to the putative MS499 plasmid found in ExPEC genomes.** Analysis of homology between the putative MS499 plasmid and ExPEC genomes with highly similar plasmids. (PDF 315 KB)

## Abbreviations

ExPEC: Extra-intestinal pathogenic *E. coli*
UPEC: Urinary tract pathogenic *E. coli*
NMEC: Neonatal meningitis-associated *E. coli*
APEC: Avian pathogenic *E. coli*
IPEC: Intestinal pathogenic *E. coli*
EHEC: Enterohaemorrhagic *E. coli*
ETEC: Enterotoxigenic *E. coli*
UTI: Urinary tract infection
ST: Sequence type
PAI: Pathogenicity island
HVH_997: *Escherichia coli* HVH 115 (4–4465997)
HVH_989: *Escherichia coli* HVH 115 (4–4465989)
GO: Gene ontology
SNP: Single nucleotide polymorphism
kbp: Kilobase pair.
