# Supplementary material for: Genomic characterisation of an endometrial pathogenic Escherichia coli strain reveals the acquisition of genetic elements associated with extra-intestinal pathogenicity
Source: BMC Genomics. 2014 Dec 6;15(1):1075. doi: 10.1186/1471-2164-15-1075 (PMC4298941; doi:10.1186/1471-2164-15-1075)
Supplement: Supplementary file 1 — Additional file 1: Comparison of the putative MS499 with similar plasmids. Comparison of the MS499 plasmid with similar plasmids found in other extra-intestinal pathogenic E. coli and Salmonella enterica isolates. (PDF 446 KB) [file 12864_2014_6792_MOESM1_ESM.pdf]

Additional file 1. Comparison of the putative MS499 plasmid with related plasmids in ExPEC and *Salmonella* isolates

a

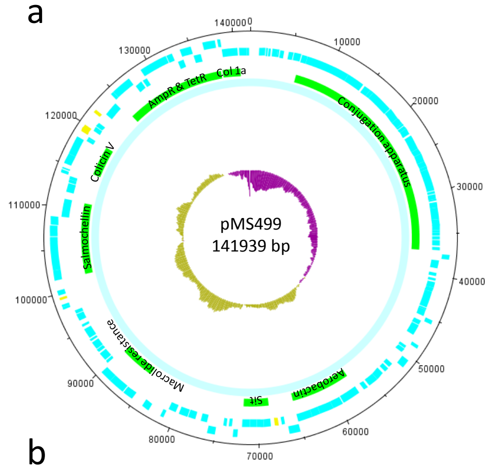

c

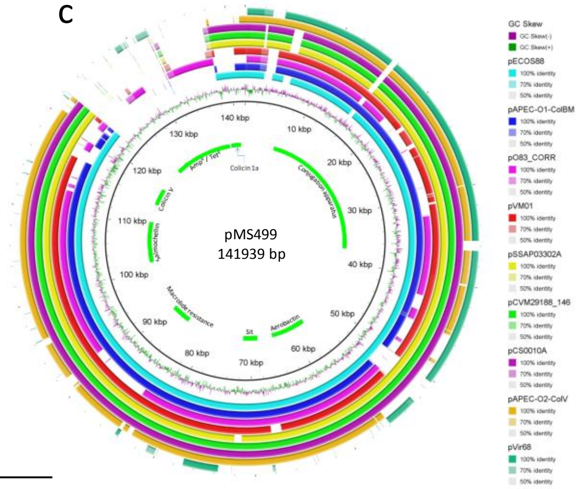

b

Feature	Genes		Function	Most similar to
Histone like protein	1	*rmoA*	Gene expression modulator	RmoA, pAPEC-O2-ColV (100% aa)
Aerobactin	5	*iucABCD,iutA*	Iron acquisition	Aerobactin locus, pCS0010A (100 % DNA)
Sit operon	4	*sitABCD*	Iron acquisition	Sit locus, pAPEC-O1-ColBM (99 % DNA)
Avian haemolysin	1	*hlyF*	Haemolysin	Several inc. HlyF, pAPEC-O1-ColBM (100 % aa)
Omptin	1	*ompP*	Outer membrane serine protease	Several inc. OmpP, pAPEC-O2-ColV (100 % aa)
Macrolide resistance operon	3	*etsABC*	Antibiotic resistance	*etsABC*, pECOS88 (100 % DNA)
Increased serum survival protein	1	*iss*	Serum resistance	Several inc. Iss, pAPEC-O1-ColBM (100 % aa)
Salmochelin	5	*iroBCDE,iroN*	Iron acquisition	Salmochelin, pCS0010A (100 % DNA)
Colicin V	4	*cvaAB,cvaC,cvi*	Bacteriocin production, export and resistance	Colicin V, pCS0010A (100 % DNA)
TEM-family beta-lactamase	1	*blaT*	Antibiotic resistance	BlaT, pSMS35_130 (100 % aa)
Tetracycline resistance	2	*tetR,tetA*	Antibiotic resistance	Several inc. pO83_CORR (100 % DNA)
Colicin 1a	2	*cipA, cia*	Bacteriocin production and resistance	Colicin 1a, pCS0010A (99 % DNA

d

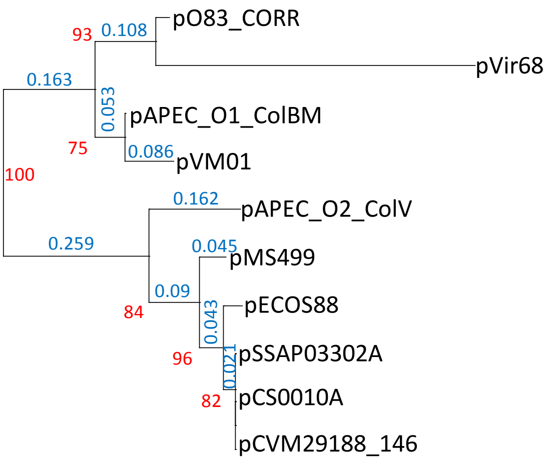

The circular MS499 plasmid is 142 kbp in length (a) and possesses several regions dedicated to putative virulence and fitness-related functions, including the Aerobactin and Salmochelin siderophore systems, the Sit iron/manganese transport system, resistance determinants for serum, macrolide, tetracycline and beta-lactam antibiotics, and two colicin loci (b). BRIG analysis shows large regions of pMS499 show striking homology to virulence plasmids found within ExPEC isolates including APEC (pAPEC-O1-ColBM, APEC-O2-ColV), S88 (pECOS88), NRG 87C (pO83_CORR), (pVM01), and several plasmids found within *Salmonella enterica* serovar Kentucky (pSSAP03302A, pCVM29188_146 and pCS0010A). In contrast, the *E. coli* plasmid pVir68 shows high levels of

homology with regions encoding plasmid replication and conjugation functions, but not with regions encoding putative virulence and fitness related functions (c). A phylogenetic analysis of the evolutionary relationship between these plasmids suggests pMS499 is most closely related to pECOS88 and plasmids found in *S. enterica* than to similar plasmids found in other *E. coli* (d).

Method

Homology between pMS499 and several other related plasmids (accession numbers listed in supplementary file 1 Table S2) was determined using the BLAST Ring Image Generator (BRIG) program [1]. Phylogenetic analysis of these plasmids was investigated by extracting the single nucleotide polymorphisms from the core genome of these plasmids using the PanSeq program [2] under the following settings: nucB: 200, nucC: 50, nucD: 0.12, nucG: 100, nucL: 20, fragmentationSize: 100, percentIdentityCutoff: 98. and constructing a maximum likelihood tree under the GTR substitution model using PhyML [3] launched within Seaview [4] with 1000 bootstrap runs.

1. Alikhan N-F, Petty N, Ben Zakour N, Beatson S: **BLAST Ring Image Generator (BRIG): simple prokaryote genome comparisons**. *BMC Genomics* 2011, **12**(1):402.
2. Laing C, Buchanan C, Taboada E, Zhang Y, Kropinski A, Villegas A, Thomas J, Gannon V: **Pan-genome sequence analysis using Panseq: an online tool for the rapid analysis of core and accessory genomic regions**. *BMC Bioinformatics* 2010, **11**(1):461.
3. Guindon S, Dufayard JF, Hordijk W, Lefort V, Gascuel O: **PhyML: fast and accurate phylogeny reconstruction by maximum likelihood**. *Infection Genetics and Evolution* 2009, **9**(3):384-385.
4. Gouy M, Guindon S, Gascuel O: **SeaView Version 4: A Multiplatform Graphical User Interface for Sequence Alignment and Phylogenetic Tree Building**. *Molecular Biology and Evolution* 2010, **27**(2):221-224.
