# Supplementary material for: Genomic characterisation of an endometrial pathogenic Escherichia coli strain reveals the acquisition of genetic elements associated with extra-intestinal pathogenicity
Source: BMC Genomics. 2014 Dec 6;15(1):1075. doi: 10.1186/1471-2164-15-1075 (PMC4298941; doi:10.1186/1471-2164-15-1075)
Supplement: Supplementary file 3 — Additional file 3: Phylogenetic analysis with bootstrap support. A subset of sequences used in our phylogenetic analysis with bootstrapping to support the branching pattern. (PDF 170 KB) [file 12864_2014_6792_MOESM3_ESM.pdf]

Additional File 3. Phylogenetic tree with bootstrap support

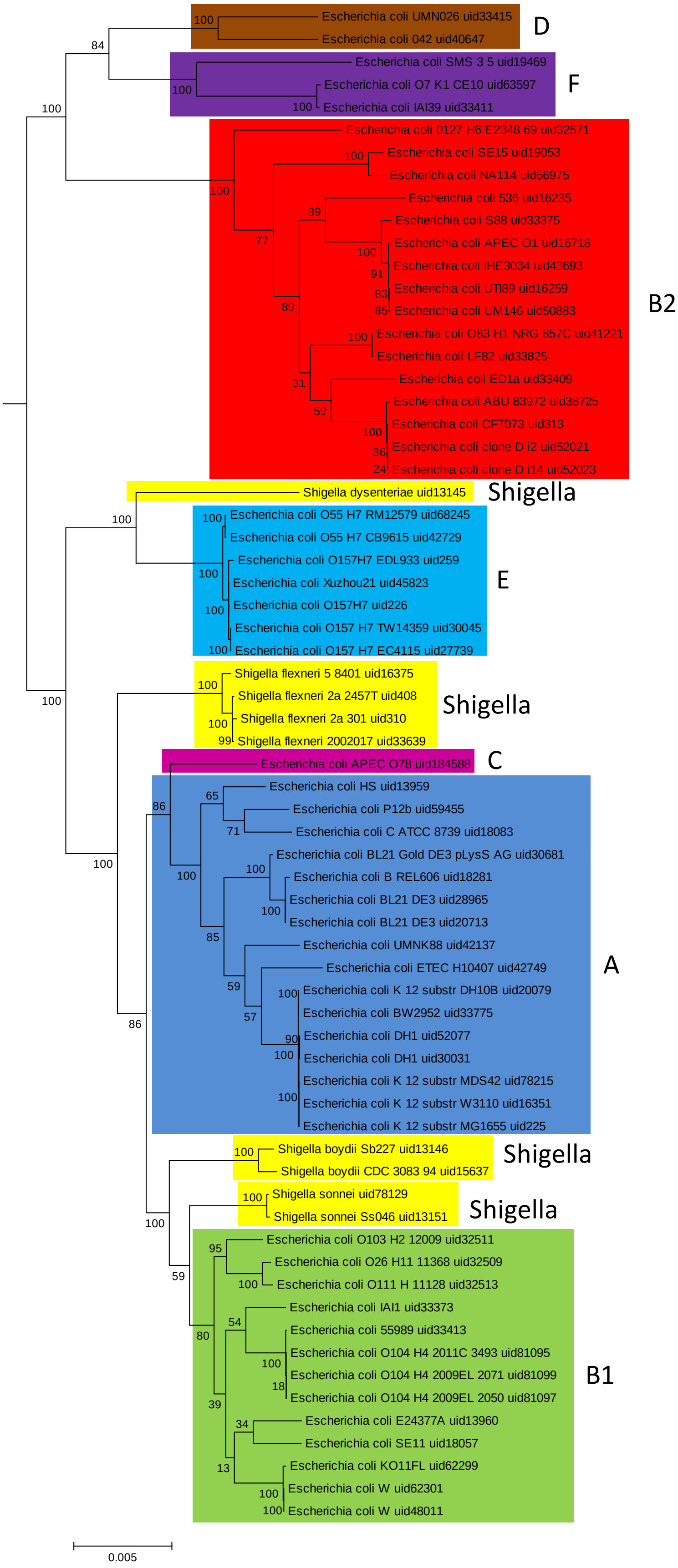

This tree shows the same principle divisions between the phylogroups as shown by the tree in figure 1 a. By using a subset of isolates, we were able to perform bootstrapping which shows that the divisions in the tree are well supported. 100 Bootstrap replicates were performed. The tips of the tree are coloured according to phylogroup, with all *Shigella* coloured yellow.
