# Supplementary material for: Genomic characterisation of an endometrial pathogenic Escherichia coli strain reveals the acquisition of genetic elements associated with extra-intestinal pathogenicity
Source: BMC Genomics. 2014 Dec 6;15(1):1075. doi: 10.1186/1471-2164-15-1075 (PMC4298941; doi:10.1186/1471-2164-15-1075)
Supplement: Supplementary file 5 — Additional file 5: The local phylogeny of MS499. A phylogenetic analysis of genomes closely related to MS499. (PDF 272 KB) [file 12864_2014_6792_MOESM5_ESM.pdf]

Additional file 5. The local phylogeny of MS499

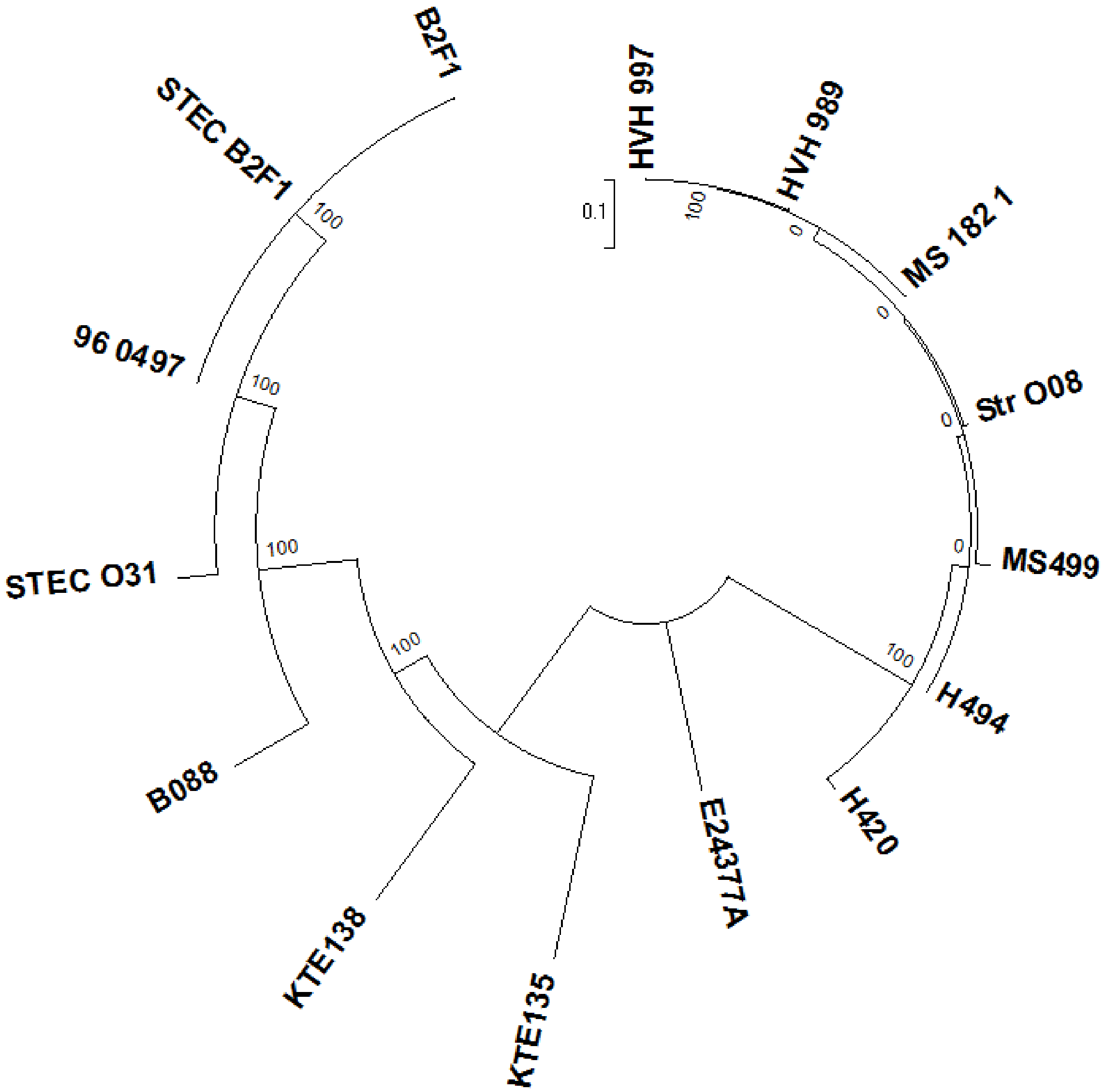

A phylogenetic reconstruction of isolates closely related to MS499, as according to our phylogenetic analysis shown in Figure 1 (main text). This tree is built from 53044 SNPs present in the genome sequence shared by all the strains, and numbers represent percent bootstrap support for 1000 replicates. These phylogroup B1 isolates fall into two clear groups, with a more distantly related group of isolates populated with several STEC *E. coli*, including B2F1 and O31, and a more closely related group of isolates including the ETEC strain E24377A, but otherwise populated with ExPECs (MS499 and Str. O08), or human commensal isolates (H494, H420, MS 182-1). However, on branches close to MS499 there is no bootstrap support for these divisions, suggesting that H494, MS499, Str. O08, MS 182-1, HVH 989 and HVH 997 are extremely closely related.

Method: Analysis of close phylogenetic relatives

To investigate the relatedness between MS499 and its closest sequenced relatives, we probed our phylogenetic data and identified 14 closely related isolates. To confirm the relationship amoung these sequence, we extracted SNPs from the core genome using the PanSeq program [1] under the following settings; nucB: 200, nucC: 50, nucD: 0.12, nucG: 100, nucL: 20, fragmentationSize: 100, percentIdentityCutoff: 98 and constructed a maximum likelihood tree under the GTR substitution model using MEGA 5.0 [2] with 1000 bootstrap runs.

1. Laing C, Buchanan C, Taboada E, Zhang Y, Kropinski A, Villegas A, Thomas J, Gannon V: **Pan-genome sequence analysis using Panseq: an online tool for the rapid analysis of core and accessory genomic regions**. *BMC Bioinformatics* 2010, **11**(1):461.
2. Tamura K, Peterson D, Peterson N, Stecher G, Nei M, Kumar S: **MEGA5: Molecular Evolutionary Genetics Analysis using Maximum Likelihood, Evolutionary Distance, and Maximum Parsimony Methods**. *Molecular Biology and Evolution* 2011.
