# Supplementary material for: Genomic characterisation of an endometrial pathogenic Escherichia coli strain reveals the acquisition of genetic elements associated with extra-intestinal pathogenicity
Source: BMC Genomics. 2014 Dec 6;15(1):1075. doi: 10.1186/1471-2164-15-1075 (PMC4298941; doi:10.1186/1471-2164-15-1075)
Supplement: Supplementary file 7 — Additional file 7: Homologous sequences to the putative MS499 plasmid found in ExPEC genomes. Analysis of homology between the putative MS499 plasmid and ExPEC genomes with highly similar plasmids. (PDF 315 KB) [file 12864_2014_6792_MOESM7_ESM.pdf]

Additional file 7: Homologous sequences to the putative MS499 plasmid found in ExPEC genomes

We identified close homologues to all ExPEC-associated genes encoded on the putative MS499 plasmid in 14 ExPEC genomes. We then used BRIG [1] to visualise the degree of homology to the MS499 plasmid present in these strains. These results showed high levels of similarity to the MS499 plasmid in all 14 ExPEC genomes, particularly HVH 146(4-3189767) which contains homologous sequences to the entire MS499 plasmid.

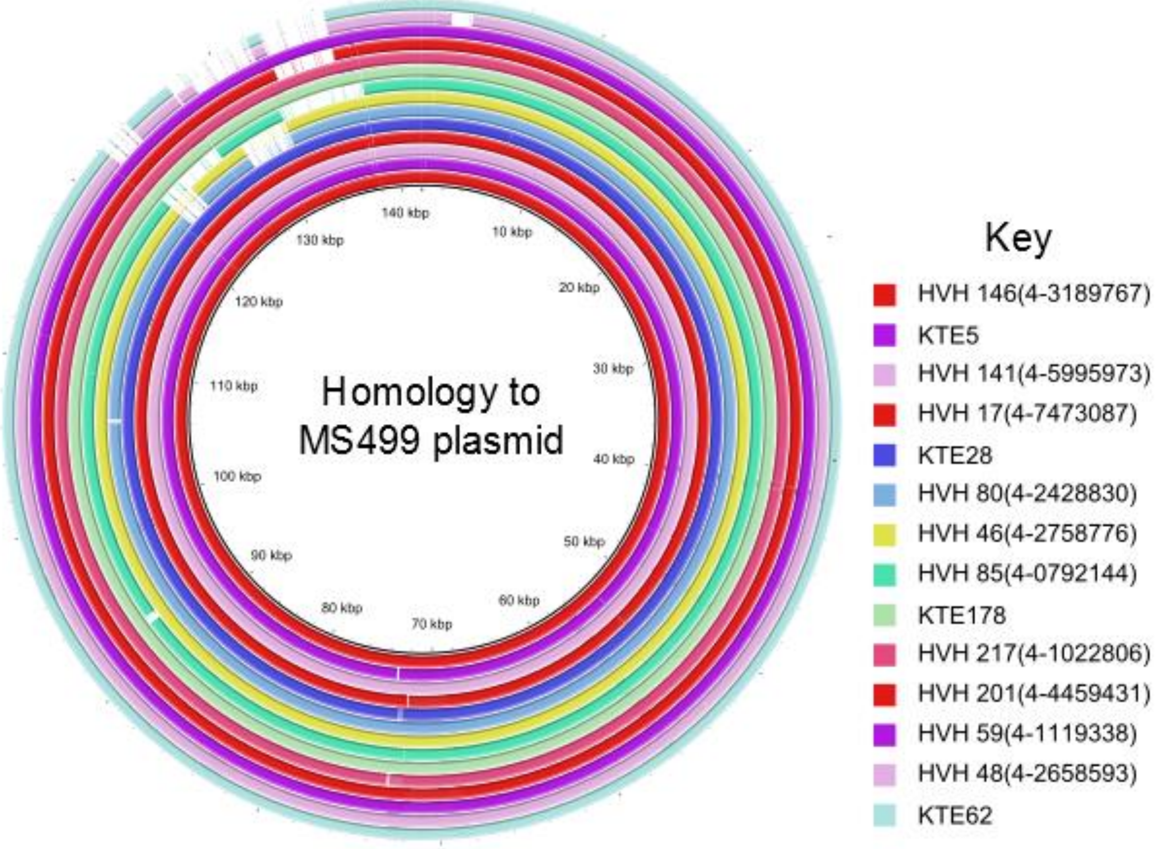

1. Alikhan N-F, Petty N, Ben Zakour N, Beatson S: **BLAST Ring Image Generator (BRIG): simple prokaryote genome comparisons**. *BMC Genomics* 2011, **12**(1):402.
